# The murine female intestinal microbiota does not shift throughout the estrous cycle

**DOI:** 10.1371/journal.pone.0200729

**Published:** 2018-07-16

**Authors:** Jessica G. Wallace, Ryan H. Potts, Jake C. Szamosi, Michael G. Surette, Deborah M. Sloboda

**Affiliations:** 1 Department of Biochemistry and Biomedical Sciences, McMaster University, Hamilton, Canada; 2 Farncombe Family Digestive Health Research Institute, McMaster University, Hamilton, Canada; 3 Department of Medicine, McMaster University, Hamilton, Canada; 4 Department of Obstetrics and Gynecology, McMaster University, Hamilton, Canada; 5 Department of Pediatrics, McMaster University, Hamilton, Canada; "INSERM", FRANCE

## Abstract

Pregnancy is accompanied by maternal physiological adaptations including metabolic, endocrine, immune, cardiovascular, skeletomuscular and neurological modifications that facilitate fetal and placental growth and development. Emerging evidence suggests that the maternal intestinal microbiota is modified over the course of healthy pregnancy. We have recently identified a maternal intestinal microbial shift within hours of conception; a shift that continued with advancing gestation. It is possible that maternal gut bacterial profiles might be associated with the known endocrine changes that accompany the female reproductive (estrous) cycle. Methods: To determine whether the estrous cycle influenced the shifts in the maternal intestinal microbiota, time-matched fecal pellets were collected daily for 3 consecutive estrous cycles from individually housed, non-pregnant female C57BL/6J mice (n = 10) fed a control diet. Estrous stage was identified by cell type predominance in vaginal cytological samples. The corresponding fecal pellets for each estrous stage were processed for bacterial 16S rRNA sequencing of the variable 3 (V3) region. Results: Estrous cycle stage accounted for a very small and not statistically significant proportion of the variation in the fecal microbiota according to PERMANOVA testing performed on Bray-Curtis dissimilarity scores. These values displayed no significant clustering of fecal microbial communities by estrous stage. Conclusion: The estrous cycle does not result in any significant shift in the intestinal microbial community in the reproductively mature, regularly cycling female mouse.

## Introduction

Approximately 10 trillion (10^13^) bacteria reside within the human intestine[1]. Collectively these microbes constitute the intestinal microbiota, and the genes they encode along with the environment they inhabit are known as our microbiome[2]. These organisms have been coined our “forgotten organ”, living symbiotically with the host, having coevolved with vertebrates over many millennia[3]. Intestinal commensals have been suggested to play a key role in the maintenance of host health: they facilitate the metabolism of indigestible polysaccharides, the production of essential nutrients, participate in energy and fat storage[4], the growth and development of the gastrointestinal system[5], the development and function of the immune system[6], the maintenance of intestinal epithelial barrier integrity[7] and protection against intestinal colonization by pathogenic bacteria[8]. More recently, it has been proposed that intestinal commensals may also influence maternal adaptation to pregnancy[9].

The intestinal microbiota is modulated by several host-related factors including genetics[10], diet[11], weight[4], age, geography[12], and health status[13]. Experimental[14] and clinical studies[9, 15] show that pregnancy is also accompanied by a shift in the intestinal microbiota. We have shown previously that pregnancy induced a shift in the maternal intestinal microbiota of lean mice, characterized by statistically significant increases in the relative abundance of 4 genera (*Akkermansia*, *Bifidobacteria*, *Clostridium*, and *Bacteroides*)[14]. Similar taxonomic shifts have been reported over the course of gestation in humans[9, 15]. This maternal intestinal microbial shift may be an important contributor to the known maternal metabolic adaptations that accompany pregnancy[9]; however the mechanisms driving changes in microbial abundance and composition are unknown and require further investigation. It is possible that female hormones that accompany reproductive stage cycles are involved in these changes.

Few studies have examined the relationship between the concentrations of circulating sex-steroid hormones with gut bacterial abundance; however it is reasonable to suppose such a relationship might exist. Estrogen metabolism occurs in peripheral organs where in the liver, conjugated estrogens are excreted in bile as estriol-3-sulfate-16-glucuronide. Following passage through the distal intestine they become activated through deconjugation by β-glucuronidase or sulfatase[16]. Estrogen metabolites are subsequently reabsorbed through the intestinal mucosa and enter the circulation as biologically active molecules[17], following which they are reconjugated into estriol-16-glucuronide which is then returned to the liver[18]. Steroids undergoing enterohepatic circulation are exposed to bacterial metabolism. *Escherichia coli* and *Bacteroides* species specialize in glucuronidase production[19], while *Clostridium*, *Lactobacillus*, *Eubacterium*, *Peptococcus* and *Bacteroides* are steroid-desulfating bacteria[20]. *Bacteroides* and *Clostridia* have been reported to be very active in bile acid metabolism[21], thus it has been hypothesized that these genera may be involved in the enterohepatic circulation of the female sex-steroid hormones 17β-estradiol and progesterone.

Early observations that germ free (GF) mice showed difficulty in generating colonies[22], had smaller litter sizes[23], and showed reduced levels of progesterone on day 1 of diestrous[22] were the first indicators of a relationship between intestinal bacteria and sex-steroid hormone levels. Colonization of GF mice with the feces of conventional counterparts has been shown to enhance reproductive capacity[22]. Similarly in postmenopausal women, levels of urinary estrogens have been correlated with measures of fecal microbiome richness and alpha diversity[17]. Other bacteria occasionally colonizing the gut, *Pseudomonas aeruginosa* and *Staphylococcus*, have been reported to metabolize estrogens and estrogen metabolites *in vitro*[24]. Equally, systemic measures of non-ovarian estrogens (via enterohepatic circulation) have shown that circulating estrogen is associated with fecal *Clostridia* and genera belonging to the *Ruminococcaceae* family[17]. Associations between fecal microbiota and urinary estrogen and 13-hydroxylated estrogen metabolites in postmenopausal women show that the ratio of estrogen metabolites to parents was directly associated with phylogenetic diversity and the relative abundances of the order *Clostridales* and the genus *Bacteroides*[25]. Finally, an investigation in estrogen receptor β (ERβ) knockout mice showed that ERβ, the most abundant estrogen receptor found in the colon, influences the composition of the intestinal microbiota where taxa belonging to the *Proteobacteria*, *Bacteroidetes* and *Firmicutes* phyla were altered as a function of ERβ status[26]. Collectively, these results highlight a potential link between bacterial abundance and estrogen metabolism.

To date, the mechanisms driving the intestinal microbial shifts during pregnancy in lean mice are unknown. We hypothesized that reproductive sex-steroid hormones may mediate the intestinal microbial shifts that occur throughout gestation[14]. In the present study, we investigated the composition of the fecal microbiota throughout the reproductive estrous cycle in regularly cycling, non-pregnant female mice to understand whether sex-steroid hormones may be a factor influencing the intestinal bacterial community in lean female mice. These data contribute to our understanding of microbial composition in lean female mice, and can inform future studies that investigate host-microbial interactions in females.

## Materials and methods

### Animal model

All animal procedures for this study were approved by the McMaster University Animal Research Ethics Board (Animal Utilization Protocol 12-10-38) in accordance with the guidelines of the Canadian Council of Animal Care. Ten (n = 10), 6 week-old female C57BL/6J mice (Jackson Laboratory Bar Harbor, ME, Strain 000664) were maintained in the same room with constant temperature (25°C) and a 12:12 light-dark cycle. Mice were fed a control standard diet (17% fat, 29% protein, 54% carbohydrate, 3 kcal/g; Harlan 8640 Teklad 22/5 Rodent Diet) and provided water *ad-libitum*. To prevent corprophagic intestinal microbial transfer between females[27] and within females between reproductive cycle stages, all females were housed individually in hanging metal cages throughout the experiment, where they could not access feces.

### Reproductive cycle determination and fecal collection

Vaginal epithelial cells and time-matched fecal samples were collected daily from each individually housed female (n = 10) for a total of 3 complete reproductive cycles (each cycle is approximately 4–5 days long; total 15 days). Fecal samples collected from each female on each day were immediately stored at -80°C until bacterial sequencing was performed. Estrous stage was identified in each female by the examination of vaginal cytological modifications where estrous stage was used as a non-invasive marker of female sex-steroid hormone concentrations. Vaginal cytological preparations were collected daily via a vaginal smear, obtained using a saline moist cotton swab. Cells were prepared on microscope slides and stained with Haematoxylin Stain 2-Gill (Fisher Chemical, CS-401-1D, Ottawa, Canada) for 5 minutes. Cytological preparations were allowed to air dry for 10 minutes at ambient temperature prior to cellular morphological evaluation. In order to determine reproductive cycle stage, the relative abundance of leukocytes, nucleated vaginal epithelial cells, and cornified vaginal epithelial cells in each smear were observed under a light microscope, as previously described[28].

Based on cell type predominance, each vaginal epithelial cell preparation was assigned to 1 of the 4 possible stages of the murine estrous cycle; diestrous, proestrous, estrus and metestrous[29]. As previously described[28], using the gold standard method of identifying murine estrous stage, reproductive stage was characterized based on the following properties[30]: a preparation primarily consisting of leukocytes was designated *diestrous*, a preparation consisting predominantly of nucleated epithelial cells was designated *proestrous*; a preparation characterized by a majority of anucleated cornified epithelial cells was designated *estrus*, and a preparation marked by an equal proportion of leukocytes, nucleated epithelial and anucleated cornified epithelial cells was designated *metestrous*.

Only regularly cycling females were used in sequencing analyses. Females were considered to be cycling regularly if diestrous was observed for a maximum of 3 consecutive days, proestrous for a maximum of 1 day, estrus for a maximum of 3 consecutive days and metestrous for a maximum of 1 day. Irregular reproductive cyclicity was identified by prolonged stage duration or the absence of an estrous stage, as previously described[28]. All females displaying irregular reproductive cyclicity were omitted from analyses. Following staging, the time-matched fecal pellets corresponding to established reproductive stages for a total of 2–3 consecutive estrous cycles were selected from regularly cycling females and processed for 16S rRNA V3 sequencing. In this study, 7 of the 10 females exhibited regular cycling and were used in all sequencing analyses.

### Genomic DNA extraction and 16S rRNA gene sequencing

Genomic DNA was extracted from fecal pellets as previously described[14] with minor modifications. Briefly, 0.2 g of fecal material was mechanically lysed in 800 μl of 200 mM NaPO_4_ monobasic (pH 8) and 100 μl guanidinium thiocyanate-ethylenediaminetetraacetic-Sarkosyl with 0.2 g of 0.1 mm glass beads (MoBio Laboratories Inc., Carlsbad, CA, USA). Additional enzymatic lysis was performed by adding 50 μl lysozyme (100 mg/ml) and 10 μl RNase A (10 mg/ml) and incubated for 1 hour at 37°C, followed by the addition of 25 μl 25% sodium dodecyl sulfate, 25 μl proteinase K and 62.5 μl 5 M NaCl and was incubated for 1 hour at 65°C. The resulting solution was centrifuged (12,000 g for 5 minutes). The supernatant was removed and combined with an equal volume of phenol:chloroform:isoamyl alcohol (25:24:1) in a new microcentrifuge tube. The sample was vortexed and centrifuged at 12,000 g for 10 minutes. The aqueous phase was removed and combined with 200 μl DNA binding buffer (Zymo Research, Irvine, CA, USA). This mixture was passed through a Clean and Concentrator-25 DNA column (Zymo Research, Irvine, CA, USA) according to kit directions, washed and elution was performed with ultrapure water.

PCR amplification of the variable 3 (V3) region of the bacterial 16S rRNA gene was performed on extracted DNA from each sample independently, as previously described[14]. Briefly, each reaction contained 5 pmol of primer, 200 mM of dNTPs, 1.5 μl 50 mM MgCl_2_, 2 μl of 10 mg/ml bovine serum albumin (irradiated with a transilluminator to eliminate contaminating DNA) and 0.25 μl Taq polymerase (Life Technologies, Canada) for a total reaction volume of 50 μl. 341F and 518R rRNA gene primers were modified to include adapter sequences specific to the Illumina technology and 6-base pair barcodes were used to allow multiplexing of samples as described previously[31]. DNA products of the PCR amplification were subsequently sequenced using the Illumina MiSeq platform (2x150bp) at the Farncombe Genomics Facility (McMaster University, Hamilton ON, Canada).

### Sequence processing and data analyses

The sl1p pipeline was used to process resultant FASTQ files[32]. In brief, Cutadapt[33] was used to trim primers and low quality bases, PANDASeq[34] to align paired-end reads, AbundantOTU+[35] to group reads into Operational Taxonomic Units (OTUs) based on 97% similarity, and the RDP Classifier[36] as implemented in Quantitative Insights into Microbial Ecology (QIIME)[37] against the Feb 4 2011 release of the Greengenes reference database[38] to assign a taxonomy to each OTU. Any OTU not assigned to the bacterial domain was removed, as was any OTU to which only 1 sequence was assigned. Chimeric sequences were identified and removed with the usearch61 algorithm implemented in QIIME[39, 40]. This processing resulted in a total of 3981373 reads (mean 66360 reads per sample; range: 402–124660) and 1157 OTUs. We eliminated a single sample that had fewer than 1000 reads, resulting in a mean read count of 67470 and a range of 19567–124660 reads per sample. All OTUs with a summed relative abundance of < 1% across all samples were excluded. The data set used for analysis contained 3943882 reads (mean 66850 reads per sample; range 19459–123671) and 127 OTUs.

Analyses of these data were completed using R(v. 3.3.1,[41]) in RStudio[42]. Data were curated using the phyloseq (v. 1.19.1,[43]), dplyr (v.0.7.4,[44]) and tidyr (v.0.8.0,[45]) packages. Taxon relative abundance bar charts were generated using custom R scripts and ggplot2 (2.2.1, [46]). Measures of β-diversity were computed using phyloseq’s implementation of the Bray-Curtis dissimilarity metric on relative abundances. The proportion of among-community variation explained by estrous stage and by individual was tested using the vegan(v.2.4.4, [47]) implementation of permutational multivariate analysis of variance (PERMANOVA) in the adonis function. Groupings according to Bray-Curtis dissimilarity were visualized via Principal Coordinate Analysis (PCoA) ordination, implemented in phyloseq and plotted using phyloseq and ggplot2. The effect of estrous stage was modeled on the relative abundance of the 25 most abundant taxa using the DESeq2 package[48]. The likelihood ratio test was used to determine the overall significance of estrous stage as a predictor within each taxon.

## Results

### Reproductive cyclicity

**Fig 1:**
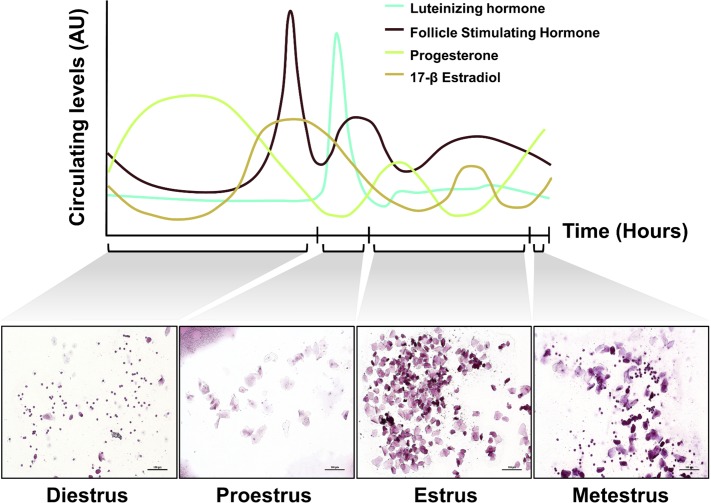
Relationship between circulating reproductive hormones (arbitaray units), reproductive cycle stage, and vaginal cytological outcomes throughout the reproductive cycle in the female mouse. Scale bar represents 100 μm.

Ten non-pregnant female mice were evaluated for reproductive cyclicity using gold standard techniques for evaluating reproductive cycle stage in rodents[30]. Each stage in the murine reproductive cycle corresponds to significant alterations in circulating sex-steroid hormones (Fig 1), thus we used reproductive stage as a non-invasive proxy to investigate the association between reproductive hormones 17β-estradiol and progesterone, and the female intestinal microbiota. Only regularly cycling females were used in the analyses. Seven of the 10 females exhibited regular reproductive cycles. This is consistent with other reports of murine female reproductive cyclicity[29]. Of the 4 stages of the reproductive cycle, the metestrous stage was insufficiently powered to perform bacterial sequencing, thus only three stages were analyzed, omitting metestrous. Of the 7 regularly cycling females, 3 consecutive diestrous, proestrous and estrus stages were identified in 6 female mice and 2 consecutive diestrous, proestrous and estrus stages were identified in the last female mouse. Thus feces were collected from n = 7 individual female mice at each estrous cycle stage for 2–3 consecutive reproductive cycles for sequencing analysis.

### The female intestinal microbiota shows no apparent shift with reproductive cycle stage

**Fig 2:**
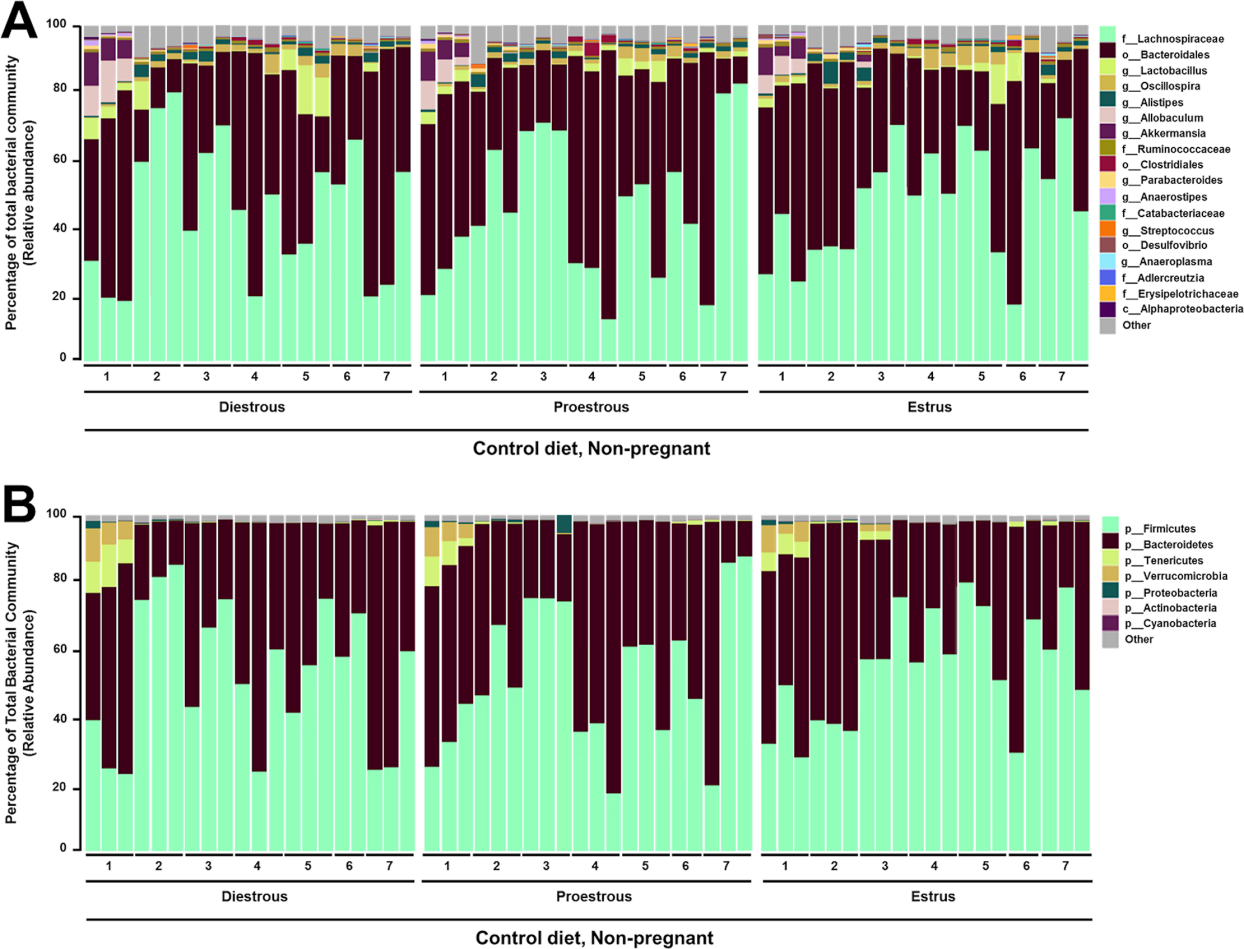
The female intestinal microbiota appears consistent throughout the reproductive cycle. **A.** Taxonomic classifications of the 25 most abundant bacterial taxa resolved to the class (c), order (o), family (f) and genus (g) level at diestrous, proestrous and estrus for 2–3 consecutive estrous cycles in female mice (n = 7). **B.** Taxonomic classifications of bacterial phyla at diestrous, proestrous, and estrus for 3 consecutive estrous cycles in female mice (n = 7).

**Fig 3:**
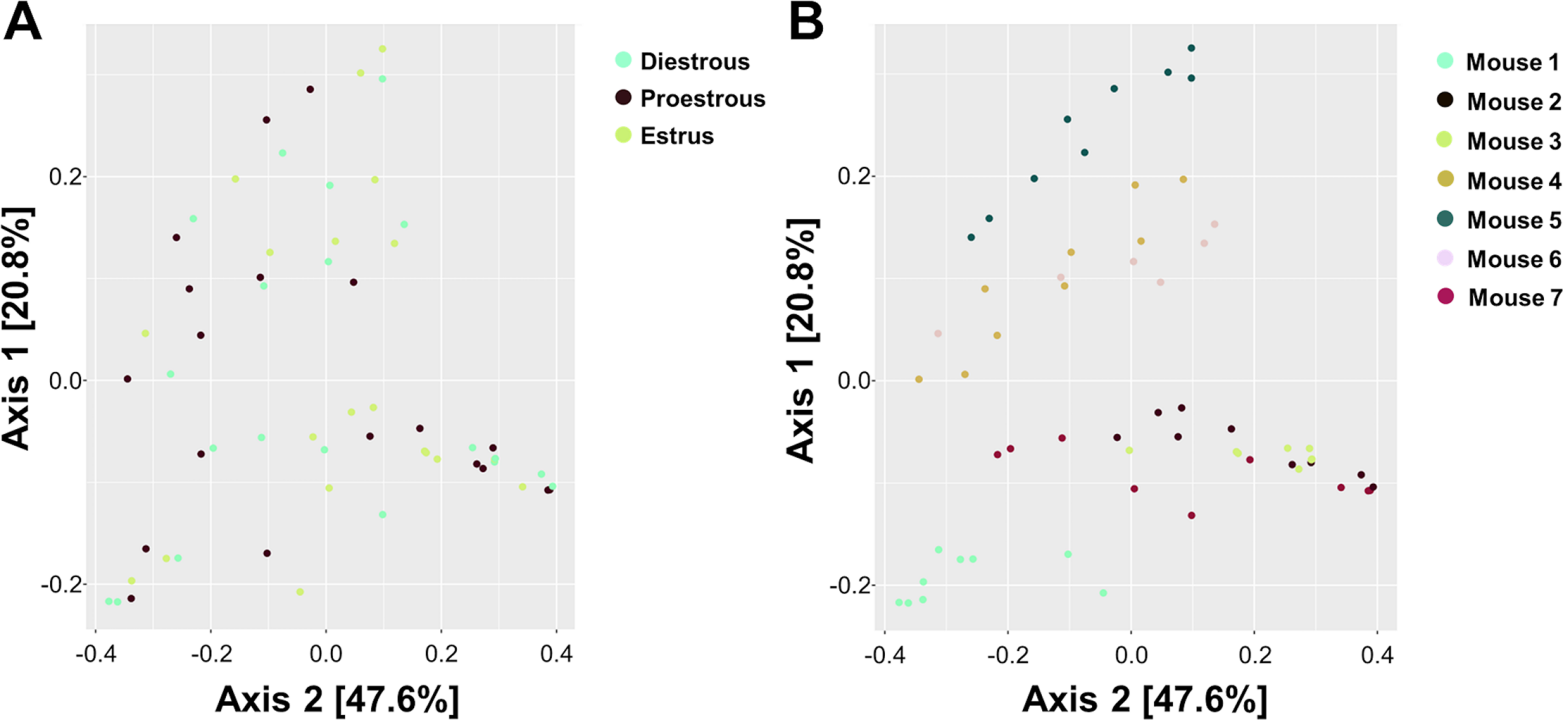
Intestinal microbial differences are driven by mouse-related differences rather than by reproductive cycle stage. **A.** Principle Coordinate Analysis using the Bray-Curtis dissimilarity metric showed no clustering of intestinal microbial communities present at diestrous, proestrous or estrus in female mice (n = 7). **B.** Principle Coordinate Analysis using the Bray-Curtis dissimilarity metric shows significant clustering of microbial communities present in each female mouse across 2–3 consecutive estrous cycles (n = 7).

There was no large or clearly observable effect of estrous stage on the fecal microbiota. The 2 dominant phyla identified in regularly cycling females were *Firmicutes* and *Bacteroidetes*, consistent with other intestinal microbial profiles reported in mice[11, 14] and humans[10]. Fig 2 displays the taxonomic summaries of microbial relative abundance at the genus (Fig 2A) and phylum (Fig 2B) levels of classification in the female intestinal microbiome at diestrous, proestrous and estrus for 2–3 consecutive reproductive cycles. No clustering of intestinal microbial communities by estrous stage was observed in the PCoA ordination of Bray-Curtis distances (Fig 3A). According to the PERMANOVA test of among-sample differences, estrous stage accounted for <1% of the total among-sample variation (PERMANOVA p = 0.79, R2 = 0.009). This propotion was not statistically distinguishable from zero. Significant clustering of the intestinal microbiota of individual female mice was found, independent of estrous cycle stage. Clustering by individual females was identified in the ordination of Bray-Curtis distances (Fig 3B) and the effect of individual according to the PERMANOVA test was significant (P = 0.001) and substantially larger (R2 = 0.594) explaining 59% of the among-sample variation. In order to further support our null findings, which suggest that the intestinal microbiota does not shift throughout the estrous cycle, we verified these findings with DESeq2. Using DESeq2, a single taxon, *Akkermansia*, was identified where estrous stage was a significant predictor of relative abundance (S1 Fig). Thus, microbial composition differs substantially more between individual female mice than between estrous stage.

## Discussion

In this study we used a non-invasive, gold standard method of establishing murine reproductive cycles in order to investigate whether female sex-steroid hormone fluctuations were associated with shifts in the intestinal microbiota. We were unable to show an association between intestinal microbial composition and reproductive stage. Our results suggest that the fecal microbiota appears consistent throughout the estrous cycle and suggest that the maternal intestinal microbial shifts that have been previously reported to occur during pregnancy[14] are likely due to factors other than the known changes in circulating 17β-estradiol and progesterone levels associated with reproductive cyclicity.

Despite the fact that steroids within the enterohepatic circulation are metabolized by gut bacteria[19, 20], little is known about the relationship between sex-steroid concentrations and intestinal microbial populations. Even less is known about whether reproductive cycles correlate with shifts in the female microbiota. Although most studies investigating sex-steroid hormones in relation to microbial shifts have concentrated on the vaginal microbiota, these reports have produced inconsistent data, where some studies show shifts in the vaginal microbiota over the course of the female reproductive cycle[49, 50], while others do not[51, 52]. To date, no association has been found to exist between circulating estradiol levels and the fecal microbiota[17]. Our study used a non-invasive approach to understand the relationship between reproductive cyclicity and populations of intestinal bacteria. An important strength of this non-invasive method is the minimization of stress, allowing for the investigation of regular reproductive cycles without this confounding factor, known to influence reproductive cyclicity in rodents[29, 30]. However, we recognize that in order to directly investigate the relationship between circulating sex-steroid levels and the female intestinal microbiota, more invasive studies that directly measure or strategically manipulate circulating sex-steroid concentrations at the time of fecal pellet collection should be performed in future investigations.

We hypothesized that pregnancy-induced changes in the maternal intestinal microbiota were mediated by reproductive sex hormones. Our present data suggests that this is not the driving factor. It is possible that factors other than female sex hormones could be orchestrating early maternal intestinal microbial shifts. Cage effects have been identified as powerful factors confounding studies investigating the intestinal microbiota[53]. In our previous study, where we observe intestinal microbial shifts at conception[14], each female mouse is co-housed with a male at the time of mating (although only for 12 hours). Thus, mating introduces a confounding variable where the female is exposed to the male cage microenvironment. Studies have demonstrated that the intestinal microbiota of individually housed mice converge to a similar profile over time following cohousing[54]. Therefore, observations of a maternal intestinal microbial shift at conception may be a representation of the male intestinal microbiota induced by female allocoprophagy of the male feces.

Based on our current data, we conclude that reproductive cycle-related sex-steroids do not have a sizable effect on the community composition of intestinal bacteria within female mice. We recognize that the feces collected may not exactly correspond with the peaks of 17β-estradiol or progesterone during the estrous cycle. To conclusively show that sex-steroids do not influence intestinal microbial shifts, serum concentrations of 17β-estradiol and progesterone should be directly measured at the time of fecal collection or fecal samples should be collected following the exogenous administration of pregnancy-related concentrations of 17β-estradiol and progesterone to non-pregnant female mice.

## Conclusions

The present study suggests that the female murine intestinal microbiota does not shift substantially over the course of the reproductive cycle in non-pregnant, regularly cycling female mice. These data contribute to our understanding of intestinal microbial communities in lean female mice and are important data to consider when evaluating studies conducted in females, on the influence of host-microbe interactions on health and disease.

## Supporting information

S1 Fig Individual bacterial taxonomic abundance at diestrous, proestrous and estrus in females. The relative abundance of the 25 most abundant bacterial taxa resolved to the order (o), family (f) or genus (g) level classification for 2–3 consecutive estrus cycles at D; diestrous, P; proestrous and E; estrus in females (n = 7).
(TIF)
