# Supplementary figures and images for: The murine female intestinal microbiota does not shift throughout the estrous cycle
Source: PLoS One. 2018 Jul 16;13(7):e0200729. doi: 10.1371/journal.pone.0200729 (PMC6047814; doi:10.1371/journal.pone.0200729)

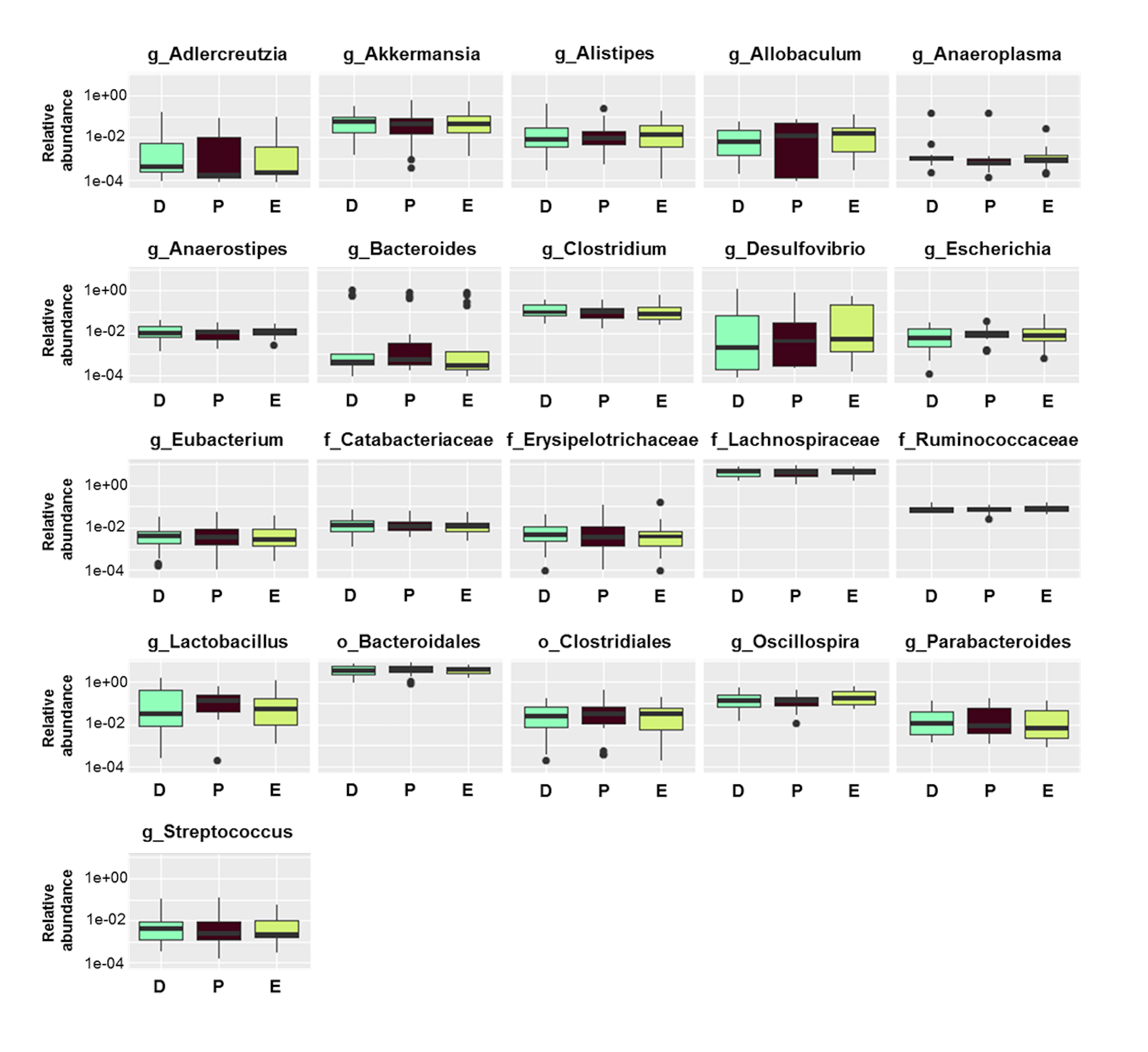

Supplement: S1 Fig — The relative abundance of the 25 most abundant bacterial taxa resolved to the order (o), family (f) or genus (g) level classification for 2–3 consecutive estrus cycles at D; diestrous, P; proestrous and E; estrus in females (n = 7). (TIF) [file pone.0200729.s001.tif]
